# CAD - CAM based 3D printed surgical guides for posterior mini-screw placement

**DOI:** 10.6026/973206300210538

**Published:** 2025-03-31

**Authors:** Sunilkumar Nagmode, Rahul Prakash Kolte, Hrishikesh Aphale, Vivek Shinde, Vinayak kalaskar, Vaishnavi Ingle

**Affiliations:** 1 Department of Orthodontics and Dentofacial Orthopaedics, SMBT dental college and hospital, Ghulewadi, Sangamner, Maharashtra, India

**Keywords:** Orthodontic mini-screws, CAD/CAM, 3D printed surgical guides, accuracy, anchorage

## Abstract

Orthodontic mini-screws provide absolute anchorage for treatments like maxillary intrusion, anterior retraction and distalization with precise placement to prevent complications. Hence, ten patients (mean age 27.8 years) were divided into two groups: direct insertion and CAD/CAM surgical guide (CS). Cone beam computer tomography (CBCT) and 3D software were used to plan mini-screw placement, with the CS group receiving 3D-printed, surgical guides for controlled insertion. Pre- and post-operative digital models were compared to assess deviations in placement. Results showed that CAD/CAM-based surgical guides enhanced accuracy by ensuring safe and predictable mini-screw insertion while minimizing complications.

## Background:

In recent years, orthodontic mini-screws have emerged as a valuable tool for achieving absolute anchorage, significantly expanding treatment capabilities and enhancing efficiency. Their application has led to improved outcomes in various orthodontic scenarios, including posterior maxillary tooth intrusion, mesial movement of teeth to close extraction spaces, and distalization of the entire maxillary arch [1-2]. Orthodontic mini-screws offer several advantages. They provide excellent mechanical retention and are particularly effective in non-compliant patients, thanks to their robust anchorage. Additionally, mini-screws are minimally invasive, relatively simple to insert and remove and cost-effective [3-4]. The widespread use of mini-implants has heightened the need for precise placement and enhanced retention. Accurate placement of mini-implants is crucial for ensuring both safety and effective absolute anchorage [4-5]. Mini-implants are frequently placed in the inter-radicular spaces of the maxillary and mandibular arches. These inter-radicular areas, especially in the posterior maxilla and mandible, are preferred for implant insertion to minimize the risk of root damage and to enhance the horizontal component of the applied force [6]. Baumgartel and Hans observed that the buccal cortical bone is thinnest in the anterior sextants of both jaws, with a progressive increase in thickness toward the posterior region. However, this trend does not apply distal to the maxillary second molars, where the buccal cortex is relatively thin [9]. In the maxillary posterior region, inserting mini-screws more than 8-11 mm above the gingival margin is generally discouraged to avoid damage to the sinus and the tuberosity area, which has limited bone and the presence of wisdom teeth. The palatal site is often recommended for implant placement over the buccal side. In the maxilla, screws are typically inserted at a 30°-40° angle to facilitate longer screw insertion. In the mandible, the safe zones for implant placement are between the 1st and 2nd molars and between the 1st and 2nd premolars [11]. Improper positioning of mini-implants can hinder the required tooth movement, thereby limiting the effectiveness of skeletal anchorage. Factors such as vertical and sagittal placement, as well as proximity to dental roots, significantly influence the stability and failure rates of mini-implants. Insertion techniques should aim to maximize the use of available bone while avoiding adjacent anatomical structures, including dental roots, naso-maxillary cavities and neurovascular tissues [10-11]. Complications resulting from incorrect placement of mini-implants can include alveolar bone fractures, root hypersensitivity or fractures, maxillary sinus perforation, and damage to the inferior alveolar nerve. Mini-implants in contact with roots are particularly prone to failure. Wu *et al.* reported that improper screw placement, without an accurate surgical guide, results in a 20% incidence of injuries during positioning. Accidental impingement of mini-implants into dental roots and periodontium can halt tooth movement for 3-4 months. Therefore, precise placement of implants is crucial for their success where "implant guides" are employed to ensure accurate placement.

## Methodology:

This split-mouth *in vivo* study was conducted in the Postgraduate Orthodontics Department. A power analysis using G*Power 3.0.1 determined a total sample size of 20 (10 per group) to achieve 80% power with an effect size of 1.35 and a significance level of 0.05. Convenience sampling selected 10 patients (mean age: 27.8 years, range: 16-45.5) requiring skeletal anchorage for anterior retraction or maxillary whole arch distalization. Patients were randomly assigned to the Direct Insertion (DI) group or the CAD/CAM surgical guide group. The mini-screws used in the study were taper-type, with a diameter of 1.5 mm and a length of 8 mm, featuring a 6 mm threaded body. For the 3D study, the standard tessellation language (STL) file of the mini-screw was utilized as a "virtual mini-screw." The mini-screw driver used for inserting the mini-screws had a cylindrical tip measuring 4.0 mm in diameter and 4.0 mm in height. The upper arches were scanned using CBCT (Dentri, HDxwill Co., Korea) with settings of 80 kV, 10 mA, FOV 16 cm x 14.5 cm, and a voxel size of 0.2 mm. DICOM files were imported into Romexis software for mini-screw positioning. Placement was planned to ensure safety, with coronal positioning at the midline between the lamina dura of the first molar's mesial root and the buccal bone, and sagittal positioning in the inter-radicular space between the second premolar and first molar. The planned insertion depth was 8 mm to fully embed the mini-screw in alveolar bone, and the direction and position were recorded as the "planned position".

The upper arches were also scanned using an intra-oral scanner (Helios 600) and exported as STL files. Virtual mini-screws were placed in pre-operative digital models to match the planned position using (Planmeca romexis dental software), and these were saved as "planned mini-screws." The STL file of the upper arch was fused with the CBCT image using designated implant planning software (3shape implant studio software). After segmenting the CBCT data and matching it with the STL file, the planned position of the mini-screws was determined, including the direction and insertion depth for both the coronal and sagittal planes. A mini-screw driver key, designed to guide the driver head to the desired direction and depth, was created based on the 3D direction of the planned mini-screw. This driver key was metal sleeve-free, with a width of 4.05 mm in diameter and a height of 6 mm to accommodate the mini-screw driver head and control the direction. The distance from the upper part of the mini-screw driver key to the bone surface was 7 mm. This method ensured control of the position and direction of mini-screw insertion from the outset until the mini-screws were placed into the planned position.

The surgical guides were designed in a tooth-borne shape, unilaterally. All parts of the surgical guide were designed with a 3 mm thickness for added strength. A corresponding tooth-supported stereo-lithographic surgical guide was printed using a 3D printer (Elegoo 3d printers US). To prevent bacterial contamination, the surgical guide was submerged in 1% chlorhexidine for 12 hours before mini-screw placement. Patients were instructed to rinse with a 0.12% chlorhexidine solution for 30 seconds. The surgical insertion of mini-screws was performed according to the allocated treatment plan. All surgeries were conducted by the same experienced clinician (JP).

## Direct insertion method:

After administering local anesthesia, mini-screws in the direct insertion group were inserted manually using operator guidance based on the planned position. The insertion point was located with a dental probe, measuring mesiodistal and vertical distances from the second molar's mesiobuccal cusp. Mini-screws were placed perpendicular to the bone and rotated slowly with light pressure until penetrating the cortical bone. The angle was adjusted to match the planned position, and insertion continued until the mini-screw was fully embedded at the planned depth.

## CAD/CAM based 3D printed surgical-guided procedure:

For the CS group, the CAD/CAM surgical guides were designed and fabricated individually to ensure accurate mini-screw placement. The guide was secured in the upper arch using the patient's bite force. After the mini-screw was attached to the screwdriver head, it was inserted into the driver key until the tip of the mini-screw made contact with the bone surface. The screwdriver head was fully covered by the driver key and securely fitted, allowing the driver key to precisely control the driver head in 3D. The operator turned the driver slowly until the mini-screw body was embedded in the alveolar bone, as indicated by a marker on the driver tip. All patients were instructed to clean the mini-screw with a toothbrush after meals. Additionally, they were advised to use a 0.12% chlorhexidine solution to rinse twice daily for 7 days.

After mini-screw insertion, the upper arch was scanned using the same intra-oral scanner (IOS) and exported to post-operative digital models. The heads of the scanned mini-screws were separated and aligned with the reference to the heads of the virtual mini-screws using the best fit alignment function. The new position of the virtual mini-screws was saved as "actual mini-screws." Finally, the actual mini-screw positions were identified in the post-operative digital models. These superimposed mini-screws were saved as "actual mini-screws." Pre- and post-operative digital models were superimposed and compared using the best fit alignment function. Actual and planned mini-screw positions were measured for 3D angular and distance deviations in the coronal and sagittal planes between groups. The superimposition and deviation measurements were conducted using Geomagic Studio software. The deviations were then calculated, yielding 10 parameters.

## Data analysis plan and methods:

Data was collected for all the samples. Statistical analysis was performed by using Statistical Product and Service Solution (SPSS) version 21 for Windows (SPSS Inc, Chicago, IL). Data normally was checked by using Shapiro-Wilk test. Descriptive quantitative data were expressed in mean and standard deviation respectively. Data was managed using Microsoft Excel. Statistical analysis was carried out by using unpaired t test.

## Results:

The results demonstrated that CAD/CAM based 3D printed guides significantly improve angular and linear precision in mini-screw placement, supporting the hypothesis that they enhance safety and accuracy Table 1. The observed improvements in accuracy with CAD/CAM based 3D printed guides suggest that these tools provide precise control over the trajectory and positioning of mini-screws. The oblique insertion angle of 30°-40°, as facilitated by the guides, aligns with optimal anatomical considerations for avoiding root damage. Table 2 compares the coronal angular deviations between the Direct Insertion (DI) and 3D printed guide groups. The mean coronal angular deviation for the direct insertion group was 12.41° with a standard deviation (SD) of 1.06°, while the 3D printed guide group exhibited a significantly lower mean deviation of 4.09° (SD = 0.26°). The mean difference between the groups was 8.32° with a standard error (SE) of 0.34°. An unpaired t-test revealed a highly significant difference between the groups with a t-value of 23.954 and a p-value of <0.001, indicating that the 3D printed guide group achieved superior accuracy in coronal angular alignment.

Table 3 details the sagittal angular deviations for both the direct insertion and CAD/CAM 3d printed guide groups. The direct insertion group had a mean sagittal angular deviation of 7.86° (SD = 0.566°), whereas the CAD/CAM 3D printed guide group demonstrated a substantially lower mean deviation of 2.49° (SD = 0.26°). The mean difference between the groups was 5.37° (SE = 0.19°). The unpaired t-test result (t = 27.258) and p-value (<0.001) confirm a highly significant improvement in sagittal angular accuracy with the use of CAD/CAM based surgical guides. Table 4 highlights the differences in coronal overall deviations measured in millimeters. The direct insertion group had a mean coronal overall deviation of 1.54 mm (SD = 0.18 mm), which was significantly higher than the CAD/CAM 3D printed guide group's mean deviation of 0.59 mm (SD = 0.07 mm). The mean difference of 0.95 mm (SE = 0.06 mm) between the two groups was statistically significant, as evidenced by the t-value of 14.757 and a p-value of <0.001. This demonstrates the CAD/CAM based 3D printed guide group's enhanced precision in coronal positioning. Table 5 compares apical overall deviations across the direct insertion and CAD/CAM 3D printed guide groups. The direct insertion group recorded a mean apical overall deviation of 1.52 mm (SD = 0.22 mm), whereas the 3D printed guide group achieved a lower mean deviation of 0.68 mm (SD = 0.07 mm).

The mean difference of 0.84 mm (SE = 0.07 mm) was statistically significant, supported by an unpaired t-test value of 11.361 and a p-value of <0.001. These results highlight the 3D printed guide group's superior performance in minimizing apical deviations. Figure 1 (see PDF) compares the angular deviations observed in the coronal and sagittal planes for two measurement methods: Direct (DI) and CAD/CAM based 3D printed guides. The Direct (DI) method shows significantly higher deviations in both coronal (12.41 degrees) and sagittal (7.86 degrees) planes compared to the 3D printed guide method, which has lower deviations (4.09 degrees for coronal and 2.49 degrees for sagittal). This highlights the precision of the 3D printed guided method over the direct approach. Figure 2 (see PDF) illustrates the differences in coronal overall and apical overall deviations between the Direct (DI) and CAD/CAM based 3D printed guide measurement methods. The Direct (DI) method exhibits higher values for both coronal overall deviation (1.54) and apical overall deviation (1.52). In contrast, the CAD/CAM based 3D printed guide method displays significantly lower deviations (0.59 for coronal and 0.68 for apical), reinforcing its accuracy in maintaining lower deviations compared to the Direct approach.

## Discussion:

This study evaluated the accuracy of CAD/CAM-based 3D printed surgical guides for posterior mini-screw placement in the maxilla, comparing them with direct insertion (DI) methods. The results demonstrated that CAD/CAM guides significantly improve angular and linear precision in mini-screw placement, supporting the hypothesis that they enhance safety and accuracy. Specifically, the CAD/CAM method showed reduced coronal and apical deviations, making it a reliable tool for minimizing root proximity risks and ensuring stable anchorage. The observed improvements in accuracy with CAD/CAM guides suggest that these tools provide precise control over the trajectory and positioning of mini-screws. The oblique insertion angle of 30°-40°, as facilitated by the guides, aligns with optimal anatomical considerations for avoiding root damage, as highlighted by Kuroda *et al.* (2007) [11]. This aligns with the findings of Jariyapongpaiboon *et al.* [2] who emphasized the utility of CAD/CAM guides in maintaining accurate angular and distance deviations for IZC mini-screws. Additionally, the consistent placement above 8 mm from the alveolar crest reinforces the importance of adhering to safety zones for ensuring high success rates. These findings underscore the role of digital planning in mitigating human error and variability inherent in manual procedures. By superimposing planned and actual outcomes, this study validates that CAD/CAM technology bridges the gap between theoretical precision and clinical execution. The results of this study are consistent with findings by Antoszewska *et al.* (2009) [10], which reported high success rates of mini-screws when placed in the maxilla, especially when adhering to anatomical guidelines. However, while Antoszewska [10] emphasized factors like loading modes and surface characteristics, this study focuses on placement accuracy as the primary metric, demonstrating the advantages of CAD/CAM technology. Similarly, Poggio *et al.* (2006) [8] highlighted safe zones for mini-screw placement in interradicular areas with adequate mesiodistal and buccopalatal bone dimensions. This study builds on their findings by demonstrating that CAD/CAM guides ensure precise positioning within these zones, particularly in challenging posterior maxillary regions. Jian-chao *et al.* (2006) [12] investigated mini-screw success rates in different anatomical regions, identifying root proximity as a key risk factor for failure. The reduced deviations achieved with CAD/CAM guides in this study directly address this issue, particularly in anatomically constrained areas between the second premolar and first molar. Notably, while Jariyapongpaiboon *et al.* (2021) [2] reported deviations specific to IZC mini-screws, this study focuses on posterior mini-screws placed between the second premolar and first molar. Despite anatomical differences, both studies confirm the superior precision of CAD/CAM-guided techniques over direct insertion methods, with reduced angular and linear deviations as common outcomes. Costa *et al.* (1998) [7], who reported variability in manual placements, this study demonstrates the superiority of guided methods in ensuring consistent outcomes. The enhanced precision provided by CAD/CAM guides supports the transition from operator-dependent techniques to technology-assisted workflows. Unexpectedly, minor angular deviations were observed even in the CAD/CAM group. These could be attributed to biological factors, such as bone density variations or patient movement during placement. While these deviations did not compromise safety or success, they highlight the need for further refinement of surgical guide designs. Similar observations were noted by Park *et al.* (2001) [5], who emphasized the impact of bone quality on placement accuracy. The findings of this study have significant implications for clinical practice: CAD/CAM guides enhance precision, making them a preferred choice for high-risk areas like the posterior maxilla. They provide a systematic approach for ensuring safe inter-radicular placement, potentially reducing the learning curve for less experienced clinicians. The integration of digital planning and 3D printing technologies streamlines workflow, promoting their adoption in routine orthodontic practices.

## Limitations of the study:

## Sample size:

The study included only 10 participants (20 mini-screws), which may limit the generalizability of the findings.

## Clinical context:

The study focused solely on posterior mini-screws in the maxilla, and results may not apply to other anatomical regions.

## Short-term assessment:

Accuracy was evaluated immediately after placement. Long-term stability and clinical outcomes were not assessed.

## Operator skill:

Although standardized protocols were followed, the potential influence of operator experience on outcomes cannot be ruled out.

## Future research directions:

Expand the study to include a larger, more diverse sample size to enhance generalizability. Evaluating the long-term stability and success rates of mini-screws placed using CAD/CAM guides. Investigate the application of CAD/CAM guides in other anatomical regions, such as the mandible. Refining guide designs to further reduce angular deviations and improve ease of use.

## Conclusion:

The accuracy of CAD/CAM-based surgical guides for posterior mini-screw placement, demonstrating their superiority over the direct insertion method is demonstrated. The guided approach significantly improved angular and linear precision, minimizing deviations and ensuring safer, more accurate placement. These findings highlight the potential of CAD/CAM technology to enhance clinical outcomes, reduce operator variability and improve orthodontic treatment predictability.

## Figures and Tables

**Table 1:** Ten parameters for accuracy analysis

**No.**	**Parameters**	**Abbreviation**	**Unit**
1	Coronal angular deviation	CAD*	Degrees
2	Sagittal angular deviation	SAD*	Degrees
3	Coronal overall deviation	COD*	mm
4	Coronal lateral deviation	CLD	mm
5	Coronal mesiodistal deviation	CMD	mm
6	Coronal vertical deviation	CVD	mm
7	Apical overall deviation	AOD*	mm
8	Apical lateral deviation	ALD	mm
9	Apical mesiodistal deviation	AMD	mm
10	Apical vertical deviation	AVD	mm

**Table 2:** Coronal angular deviation (in degree) comparison

	**Mean**	**SD**	**Mean Difference (SE)**	**Unpaired t test**	**P value, Significance**
Direct (DI)	12.41	1.06	8.32	t = 23.954	p<0.001**
CAD/CAM based 3D printed guide	4.09	0.26	-0.34		
**p<0.001 - highly statistical significant difference

**Table 3:** Sagittal angular deviation (in degree) comparison

	**Mean**	**SD**	**Mean Difference (SE)**	**Unpaired t test**	**P value, Significance**
Direct (DI)	7.86	0.566	5.37	t = 27.258	p<0.001**
CAD/CAM based 3D printed guide	2.49	0.26	-0.19		
**p<0.001 - highly statistical significant difference

**Table 4:** Coronal overall deviation (in mm) comparison

	**Mean**	**SD**	**Mean Difference (SE)**	**Unpaired t test**	**P value, Significance**
Direct (DI)	1.54	0.18	0.95	t = 14.757	P<0.001**
CAD/CAM based 3D printed guide	0.59	0.07	-0.06		
**p<0.001 - highly statistical significant difference

**Table 5:** Apical overall deviation (in mm) comparison

	**Mean**	**SD**	**Mean Difference (SE)**	**Unpaired t test**	**P value, Significance**
Direct (DI)	1.52	0.22	0.84	t = 11.361	P<0.001**
CAD/CAM based 3D printed guide	0.68	0.07	-0.07		
**p<0.001 - highly statistical significant difference
